# Syphilis in Ancient and Prehistoric Times

**Published:** 1892-09

**Authors:** 


*Syphilis in Ancient and Prehistoric Times.* By Dr. F. BURET. Translated by A. H. OHMANN-DUMESNIL, M.D. Pp. xvi., 226. Philadelphia: F. A. DAVIS. 1891.

This book gives a most interesting account of the author's researches into the history of syphilis. An attempt is made, and with a considerable measure of success, to show that syphilis existed amongst the ancient races inhabiting China,

India, Egypt, Palestine, Greece, and Rome, and that the skulls and bones of prehistoric man reveal traces of its ravages, as nodes have been found on various parts of the skeleton.

The symptoms, and the treatment of the various phases and complications of the disease, are clearly enunciated, and the book altogether is a most readable one.

